# Genetic diversity assessment of *Hopea hainanensis* in Hainan Island

**DOI:** 10.3389/fpls.2022.1075102

**Published:** 2022-12-07

**Authors:** Yukai Chen, Hai-Li Zhang, Li Zhang, Mir Muhammad Nizamani, Taoxiu Zhou, Haiyang Zhang, Tingting Liu

**Affiliations:** ^1^ Ministry of Education Key Laboratory for Ecology of Tropical Islands, College of Life Sciences, Hainan Normal University, Haikou, China; ^2^ Hainan Key Laboratory for Sustainable Utilization of Tropical Bioresources, School of Life Sciences, Hainan University, Haikou, China; ^3^ Guizhou Normal University Museum, Guizhou Normal University, Guizhou, China; ^4^ Department of Plant Pathology, Agricultural College, Guizhou University, Guiyang, China; ^5^ College of Biological Science and Technology, Yangzhou University, Yangzhou, China; ^6^ College of International Studies, Sichuan University, Chengdu, China

**Keywords:** conservation biology, endangered tree, hopea hainanensis, SNP, GBS

## Abstract

*Hopea hainanensis* (Dipterocarpaceae) is an endangered tree species restricted to Hainan Island, China, and a small part of Northern Vietnam. On Hainan Island, it is an important indicator species for tropical forests. The wood of *Hopea hainanensis* has a very high utilization value in nature since it is compact in structure, hard in texture, not easily deformed after drying, durable, and resistant to sunlight and water. As a result of its high quality, it has been felled and mined by humans without restraint, resulting in a reduction of its population size, severe habitat fragmentation, and a sharp decline in its population. Therefore, its conservation biology needs to be researched urgently. Researchers are currently focusing on the ecological factors and seed germination in the habitat of *Hopea hainanensis* to determine its endangered status. In the literature, there are no systematic analyses of the endangered mechanism of *Hopea hainanensis* in terms of genetic diversity. It focuses especially on the systematic genetic diversity of *Hopea hainanensis* in fragmented habitats. Using single nucleotide polymorphism (SNP) and genotyping-by-sequencing (GBS) technology, 42 samples from seven different cohabitation groups were genotyped. The results showed that the average heterozygosity of the seven populations of *Hopea hainanensis* was 19.77%, which indicated that the genetic diversity of *Hopea hainanensis* was low. Genetic diversity research is essential for rare and endangered plant protection research. We can find a scientific basis for protecting endangered plants on slope bases by analyzing genetic differences and relationships among populations.

## 1 Introduction

The Earth’s biodiversity quickly decreases due to agricultural growth, over-exploitation, deforestation, pollution, and climate change (Wang et al., 2007). Around 40% of plant species are on the verge of extinction (Ly et al., 2018). Conservation genetics, a new science that uses population genetics principles and methods to biological conservation, aims to save endangered species from extinction (Hogarth et al., 1997; Sarath Padmanabhan and Hemaprabha, 2018). Endangered animals are frequently distinguished by tiny, scattered populations and limited gene flow among populations (Mehmood et al., 2018). Mating happens more commonly among relatives in tiny, isolated populations, and selfing may be observed in hermaphroditic plants. Inbreeding causes homozygosity in harmful recessive genes and, as a result, the generation of poorer offspring, a condition known as inbreeding depression (Kardos et al., 2016).

Furthermore, genetic drift is higher in small populations, contributing to the fixation of harmful mutations and loss of genetic variation, weakening a population’s adaptive ability and raising its extinction risk (Kardos et al., 2021). The area of conservation genetics, which aims to research the genetic diversity, population differentiation, mating system, and historical demography of endangered species, gives amazing insights into preserving biodiversity in the real world (Brown et al., 1991). Furthermore, *Hopea hainanensis* research is primarily concerned with the impacts of various environmental conditions in the habitat on seed germination and seedling development, *ex situ* conservation, and better seed selection and cultivation techniques in artificial propagation and cultivation (Zhang et al., 2022).

Dipterocarpaceae comprise 20-50% of the basal forest area and more than 50% of the canopy trees in tropical Asian forests (Ghazoul, 2016). Because many Dipterocarpaceae species are valuable wood resources, they have been widely exploited in tropical Asian nations. As a result of the widespread harvesting of timber and destruction for agriculture, many dipterocarps are now designated as endangered or severely endangered (Wang et al., 2021). On the other hand, Dipterocarp woods are considerably more than just a supply of lumber. They are vital components of Asian tropical rainforest ecosystems, acting as the foundation for these varied ecosystems. Indeed, Southeast Asia is home to four of the world’s 25 “biodiversity hotspots” (Wang et al., 2020). Furthermore, dipterocarp forests provide various ecosystem services and play a significant role in balancing ecological processes at the regional and global levels (Agoramoorthy, 2002). It is the representative and endemic species of the tropical ravine rainforest in Hainan.

The natural survival population of *H. hainanensis* in Hainan is mainly distributed in the forest patches dominated by broken secondary rainforests in and around Limushan, Bawangling, Jianfengling, Diaoluoshan, Yinggeling, Wuzhishan, and other forest areas in Hainan Island (Song et al., 2020). *H. hainanensis* is listed as a class I protected plant in the List of China’s National Key Protected Wild Plants (Jiang, 2019). It was identified as an endangered species in the Red Book of Chinese Plants and is ranked as “Endangered” by the IUCN (Ly et al., 2018). Currently, studies on *H. hainanensis* mainly focus on the effects of various environmental factors in the habitat on seed germination and seedling development, *ex situ* protection, and improved seed selection and cultivation techniques in artificial propagation and cultivation (Chen et al., 2015). There is a lack of systematic analysis of the endangerment mechanism in terms of genetic diversity, and there is even less research on the systematic genetic diversity of ports in different fragmented and chemical habitats (Li et al., 2015; Zhang et al., 2022).

However, genetic diversity as an extinction mechanism for *H. hainanensis* has not been systematically studied. A few studies have been conducted on the genetic diversity of *Hopea hainanensis* systems in fragmented and metaplastic environments. Wang et al. isolated and identified 12 polymorphic microsatellite markers on endangered *H. hainanensis* (Wang et al., 2020). The genetic diversity and population structure of 10 H. hainanensis populations were analyzed using 12 SSR markers in Hainan Island. The emergence of population bottlenecks may cause genetic diversity loss and population differentiation. Long-term protection strategies for endangered species in Hainan were proposed.

In many fields, genotyping by sequencing (GBS) in simplified genome sequencing technology has become increasingly popular as next-generation high-throughput sequencing technology has developed (Mehmood et al., 2022). These include the construction and analysis of genetic maps, the study of genome-wide association systems and gene diversity and identifying the germplasm of plants and animals. Therefore, in this study, GBS technology was used to systematically identify 42 genome-wide SNPs of H. hainanensis resources. Based on the identified SNPs, a phylogenetic tree of these 42 *H. hainanensis* resources was constructed, and genetic diversity was analyzed. It has practical guiding significance for the protection of *H. hainanensis* resources and the improvement of its ecological environment. It is of great significance to the protection of *H. hainanensis* biodiversity.

## 2 Materials and methods

### 2.1 Study area

**Figure 1:**
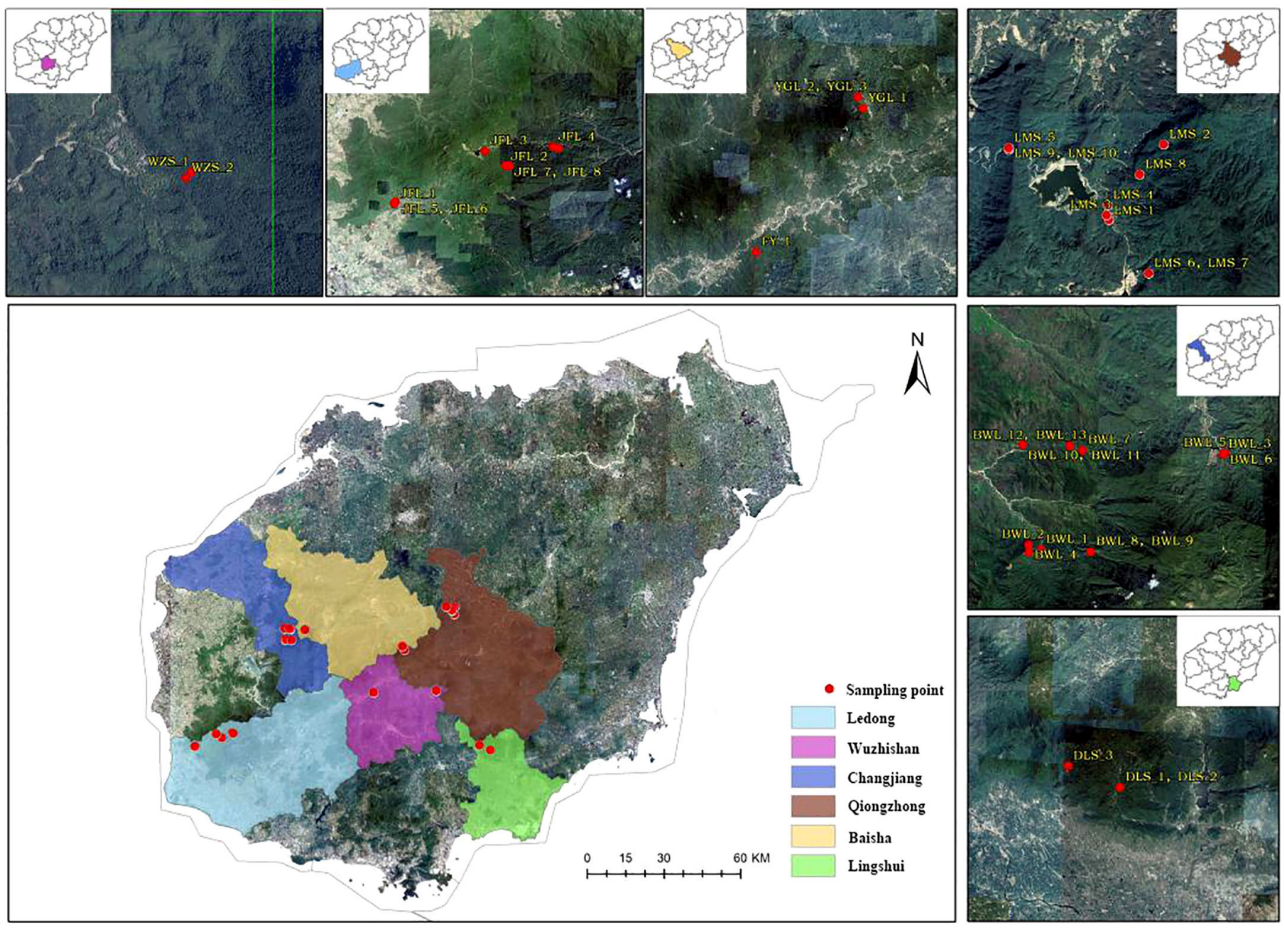
A distribution and sample collection information map of *Hopea hainanensis* in Hainan Island.

Hainan Island (E108°37′-111°03′, N18°10′-20°10) is located in southern China (Zhang et al., 2021), and it is the largest island city in China (**Figure 1**). Hainan Island has a mild climate, with an annual average temperature of 22-27°C, and is rich in forest resources (Zhang et al., 2022). Hainan Island is low and flat all around and towering in the middle, with Wuzhishanand Yinggeling as the uplift cores and descending step by step to the periphery. (https://www.hainan.gov.cn). Hainan Island is hailed as the largest “natural museum” by biologists, and 102 rare animals in Hainan Island have been included in the list of national first- and second-class key protected animals (Zhang et al., 2022). There are many rare and endangered wild plants. At the same time, northern and coastal regions have relatively low biodiversity and fewer rare and endangered species due to greater human disturbance intensity (Nizamani et al., 2021).

### 2.2 Materials

In the early field investigation, field investigation and actual sampling possibilities are based on the natural distribution of the levee population. The *H. hainanensis* sample materials were divided into seven regional populations according to their geographical sources for population sampling. The bawangling population includes nine subpopulations, namely BWL P1-P9, including samples BWL1-BWL_13. Limushan population consists of 7 subpopulations, LMS P1 and LMS P3-P8, including samples LMS1-LMS_10, respectively. The Jianfengling population included seven subpopulations JFL X1-X2, JFL P1, JFL P3-P6, which included samples JFL1-JFL_10. Wuzhishan population included two subpopulations (WZS P1-P2), including samples WZS_1-WZS_2, respectively. Yinggeling population included two subpopulations YGL P1-P2, including YGL1-YGL_3, respectively. The Diaoluoshan population included one subpopulation, DLS P1, including samples DLS_1-DLS_3. Fanyang population included one subpopulation FY1, including sample FY_1. The Fanyang population belongs to the Wuzhishan region. Regional population division, longitude, latitude, and sample number are shown in **Table 1**.

**Table 1:** The list of information for *Hopea hainanensis* sample collection in Hannan Island.

Area code	Location	Longitude	Latitude	Sample	Number of sample
BWL	Bawanglin	109°03′-109°17′E	18°57′-19°11′N	BWL_1-BWL_13	13
LMS	Limushan	109°39′-109°48′E	19°07′-19°14′N	LMS_1-LMS_10	10
JFL	Jianfengling	108°44′-109°02′E	18°23′-18°52′N	JFL_1-JFL_10	10
WZS	Wuzhishan	109°39′-109°47′E	18°49′-18°58′N	WZS_1-WZS_2	2
YGL	Yinggeling	109°11′-109°34′E	18°49′-19°08′N	YGL_1-YGL_3	3
DLS	Diaoluoshan	109°11′-109°34′E	18°49′-19°08′N	DLS_1-DLS_3	3
FY	Fanyang	109°27′E	18°53′N	FY_1	1

### 2.3 Methods

#### 2.3.1 Sample DNA extraction and quality testing

42 *H. hainanensis* leaf samples were extracted with a Biotech (Beijing, China) DNA extraction kit (Plant no. DP305) under the standard operating procedure. After DNA extraction, the quality and concentration of DNA samples should be tested. Qubit was used to determine the concentration of DNA samples, and AGE was used to detect the quality of DNA samples (Wang et al., 2015).

#### 2.3.2 Construction and sequencing of genomic GBS library

The quality and concentration of the extracted DNA were tested to be qualified and then sent to Hangzhou Lianchuan Biotechnology Co., Ltd. for GBS library construction and sequencing. RsaI-HaeIII digestion was used for digestion. The high-throughput sequencing library was prepared through terminal repair, A-tail addition, sequencing adaptor addition, purification, PCR amplification, and so on. The library was purified by electrophoresis and gluing according to the preset scheme. The gluing range of the library was 450-500bp to select the library with the length of the inserted fragment in the target interval for subsequent sequencing. Only libraries that had been screened for fragment length were qualified for sequencing. The sequencing platform was Illumina Nova6000, and the sequencing mode was PE150.

#### 2.3.3 SNP mining in *H. hainanensis* genome

After the sequencing data is taken off the machine, the raw read data obtained by sequencing is quality-controlled, and low-quality sequences and splice sequences are removed to obtain a clean read. After that, all samples’ reads are clustered, similar reads are clustered together as a tag, and the consensus sequence is generated. Then, the data were aligned with the consensus sequence by the individual, and the Clean Read data were aligned with the consensus sequence. GATK software and SAMTOOLS software were used for SNP detection, and the quality filtering of the detected mutation sites was carried out. The evolutionary analysis was based on SNP data. Before evolutionary population analysis, SNPs were filtered according to the minor allele frequency (MAF) > 0.05 and data integrity > 80% (i.e., no more than 20% of individual genotypes were missing).

#### 2.3.4 Phylogenetic tree analysis of *H. hainanensis*

The phylogenetic tree is a diagram used to describe the genetic differentiation relationship between species. According to the evolutionary relationship between different sources and different types of organisms, all kinds of organisms are placed on the branching tree diagram. The evolution process and the relationship between these organisms are succinctly described. Based on SNP, 1000 replicates of the PDIST model were obtained as phylogenetic trees of all samples based on the neighbor-joining algorithm of MEGA software (Dieckmann et al., 2016).

#### 2.3.5 Principal component analysis

Principal Components analysis (PCA) was performed based on SNP to obtain the clustering of Principal components of all samples. Through PCA analysis, it can be known which samples have short genetic distances and close relatives. The samples with long genetic distances and distant relatives are helpful for population genetic evolution analysis.

#### 2.3.6 Analysis of population genetic structure

Population genetic structure analysis can provide information on the origin and composition of individual lineage. Based on SNP, the population structure of all samples was analyzed by ADmixture software, and the number of clusters (K value) was assumed to be 1-10, respectively. Different K values represent the distribution of ancestral genetic material of different populations under the assumption that there are K ancestral groups.

#### 2.3.7 Analysis of the genetic relationship

Based on SNP, the genetic distance between pairs of all samples was calculated. We can get the relative distance between samples by analyzing genetic distance data, which can assist the evolution analysis. In the phylogenetic heat map, the redder the color, the closer the relationship between the two individuals on the horizontal and vertical axes, the large area of red in the phylogenetic heat map between multiple individuals indicates that these individuals may constitute a closely related family group. Conversely, the bluer the heat map, the more distant the relatives.

## 3 Results

### 3.1 The quality of sequencing

After GBS sequencing, 28.09 Gb of Raw READ data were obtained from 42 *H. hainanensis* samples. After removing low-quality sequences, sequences containing more than 5% N (N represents undetermined base information), and adapter sequences, 27.85 Gb of high-quality sequencing data (Clean data) was obtained. The average size of each sample is 0.66 Gb. The average proportion of base error rate below 1% (Q20) was 96.66%, and the average proportion of base error rate below 0.1% (Q30) was 91.34%, indicating the high quality of sequencing. The average ratio (GC content) of guanine (G) and cytosine (C) among the four bases of DNA was 47.84%, indicating that the distribution was reasonable. The data overview of each sample is shown in **Table 2**.

**Table 2:** The materials used in this study and overview of the GBS dataset.

Sample	Raw data (bp)	Raw data	Clean data (bp)	Clean data	Effective data (%)	Q20 (%)	Q30 (%)	GC (%)
BWL_1	3889430	0.58G	3840274	0.57G	98.5	96.77	91.59	50.46
BWL_2	4353376	0.65G	4307204	0.64G	98.77	96.97	92	47.31
BWL_3	4410548	0.66G	4361182	0.65G	98.67	96.65	91.3	48.35
BWL_4	5189486	0.78G	5136018	0.77G	98.78	96.97	91.97	47.43
BWL_5	4267498	0.64G	4218714	0.63G	98.63	96.74	91.53	47.6
BWL_6	4538054	0.68G	4487112	0.67G	98.67	96.75	91.5	47.63
BWL_7	4157322	0.62G	4111528	0.62G	98.68	96.68	91.35	47.67
BWL_8	4475100	0.67G	4426518	0.66G	98.71	96.66	91.3	47.72
BWL_9	4937116	0.74G	4886146	0.73G	98.81	96.68	91.31	47.43
BWL_10	4079886	0.61G	4027066	0.60G	98.5	96.47	90.94	47.86
BWL_11	4272788	0.64G	4224600	0.63G	98.64	96.62	91.23	47.32
BWL_12	3689286	0.55G	3652654	0.55G	98.8	96.89	91.79	47.31
BWL_13	5344202	0.80G	5276592	0.79G	98.52	96.61	91.26	47.54
LMS_1	4132158	0.62G	4078226	0.61G	98.48	96.79	91.65	47.51
LMS_2	4568574	0.69G	4503136	0.67G	98.39	96.3	90.59	49.58
LMS_3	4821746	0.72G	4757702	0.71G	98.48	96.6	91.24	48
LMS_4	4205644	0.63G	4159754	0.62G	98.71	96.84	91.7	49
LMS_5	5239770	0.79G	5175988	0.77G	98.58	96.7	91.44	47.39
LMS_6	4629342	0.69G	4577802	0.69G	98.69	96.89	91.83	47.7
LMS_7	4531974	0.68G	4476688	0.67G	98.59	96.7	91.45	48.19
LMS_8	4704600	0.71G	4654786	0.70G	98.75	96.76	91.49	47.3
LMS_9	4455698	0.67G	4406112	0.66G	98.68	96.77	91.55	47.45
LMS_10	3780250	0.57G	3741060	0.56G	98.75	96.83	91.67	47.82
JFL_1	4217110	0.63G	4166782	0.62G	98.61	96.64	91.27	47.93
JFL_2	5198358	0.78G	5130962	0.77G	98.53	96.46	90.9	47.97
JFL_3	4385206	0.66G	4324702	0.65G	98.44	96.4	90.79	47.67
JFL_4	2491648	0.37G	2453514	0.37G	98.24	96.34	90.71	47.39
JFL_5	4668386	0.70G	4607144	0.69G	98.54	96.26	90.48	47.9
JFL_6	6462998	0.97G	6396564	0.96G	98.8	96.84	91.63	46.6
JFL_7	2447580	0.37G	2423086	0.36G	98.69	96.78	91.57	48.35
JFL_8	5394202	0.81G	5330052	0.80G	98.61	96.71	91.43	47.75
JFL_9	5101784	0.77G	5043916	0.76G	98.67	96.77	91.57	47.92
JFL_10	6088498	0.91G	6023038	0.90G	98.76	96.81	91.6	47.54
WZS_1	4060186	0.61G	4003372	0.60G	98.42	96.69	91.45	47.78
WZS_2	6086906	0.91G	6017660	0.90G	98.67	96.84	91.67	47.33
YGL_1	4555380	0.68G	4505358	0.67G	98.74	96.89	91.82	48.3
YGL_2	4127066	0.62G	4066052	0.61G	98.3	96.18	90.33	47.46
YGL_3	3659212	0.55G	3615938	0.54G	98.63	96.43	90.84	47.64
DLS_1	3968904	0.60G	3921372	0.59G	98.54	96.8	91.64	48.71
DLS_2	3803398	0.57G	3754674	0.56G	98.5	96.49	90.98	48.3
DLS_3	4899390	0.73G	4838058	0.72G	98.58	96.71	91.44	48.09
FY_1	3039210	0.46G	2882090	0.43G	94.63	96.19	90.57	47.28

### 3.2 SNP site mining

After comparing the data to the consensus sequence, GATK and SAMTOOLS software were used for mutation detection (Wright et al., 2019). SNPs consistently output by the two software were retained as reliable loci. According to the criteria of MAF >0.05 and data integrity >0.8, SNP data were further processed and filtered to retain SNP_S_ with polymorphisms. After filtering the SNPs obtained, 430376 high-quality SNPs were finally obtained for subsequent analysis. It can be seen from the following **Table 3** that the heterozygosity of the Fanyang population (FY) is relatively high, which may be related to the fact that the Fanyang population has only one sample, the sample size is small, the width of the genetic variation is insufficient and other factors, so there is not enough sample data for comparative analysis of the genetic diversity in this population. The heterozygosity of the other six populations ranged from 19.26% to 20.34%, with average heterozygosity of 19.77%, indicating a low level of genetic diversity.

**Table 3:** SNP Statistical results.

Population code	SNP number	Heter LociNum	Homo LociNum	Hetloci-ratio
BWL	84652	16895	67757	19.96%
LMS	99347	19073	80275	19.26%
JFL	86427	17578	68849	20.34%
WZS	92887	18325	74562	19.73%
YGL	80498	15682	64816	19.48%
DLS	86604	17195	69409	19.85%
FY	17510	9285	8225	53.03%

### 3.3 Genetic evolution and population analysis

#### 3.3.1 Phylogenetic evolutionary tree

**Figure 2:**
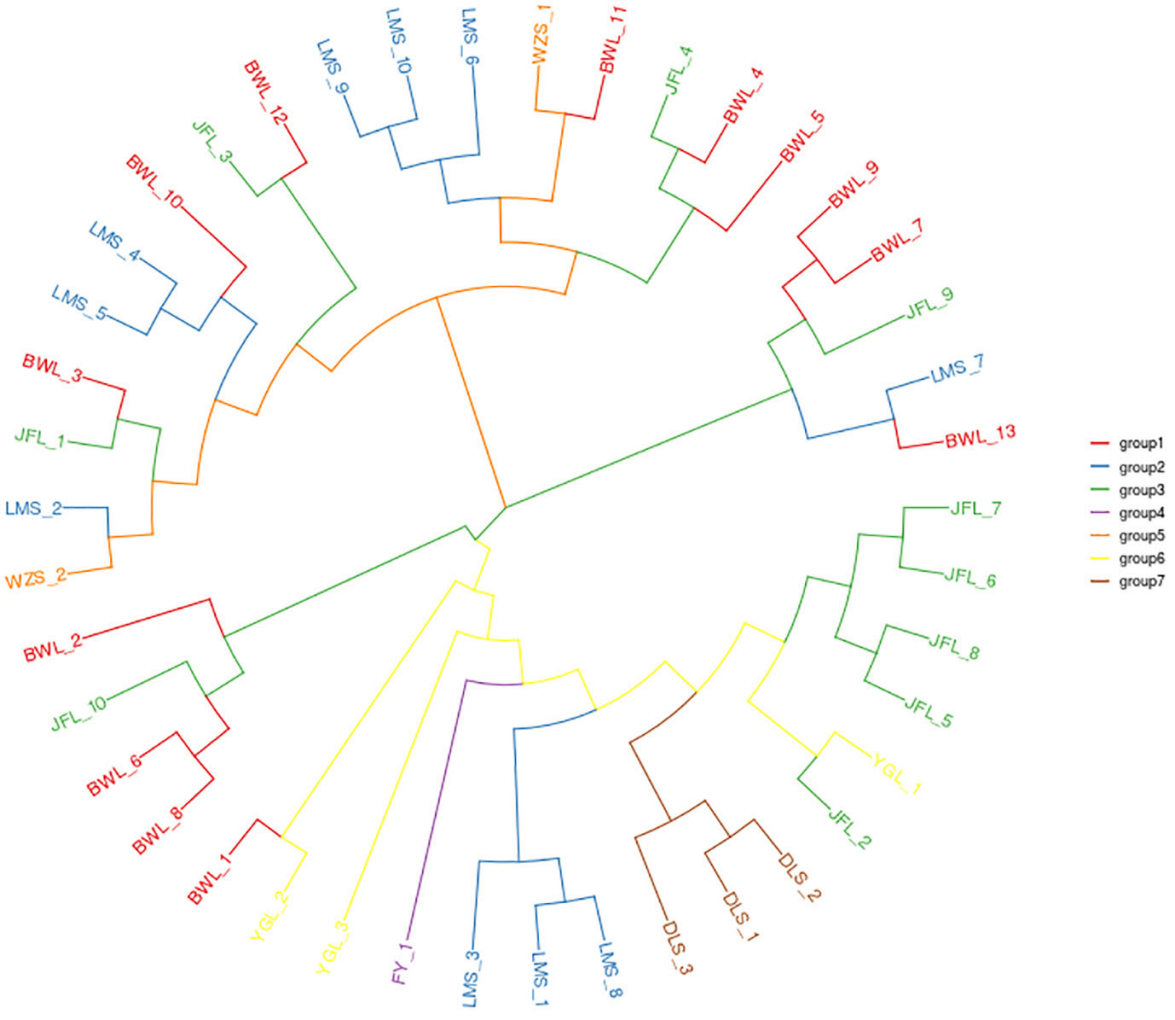
The neighbor-joining clustering of *Hopea hainanensis* in different Population.

The identified high-quality SNPs were used for phylogenetic analysis of the 42 *H. hainanensis* resources. After 1000 repetitions based on the PDIST model, the neighbor-joining algorithm of MEGA software was used to perform evolutionary analysis on all samples, and the phylogenetic tree of 42 *H. hainanensis* sample resources was obtained (**Figure 2**). Samples from the same sampling site were relatively closer to each other. However, the relative distance between the samples from different sites means that the internal samples from different sampling sites in these seven population areas may have a common ancestor. The results showed that the 42 samples could be divided into two large groups, and each could be further divided into small subgroups. In general, the samples of the same geographical origin were relatively aggregated in the two large taxa. Still, the distribution was mixed in the small subgroups, and the samples of the same geographical origin were not completely merged into the same subgroup. Group I mainly include the resources from Diaoluoshan, Fanyang Mountain, and Yinggeling, and the resources from Wuzhishanare clustered into Group II. The resources from Limushan, Bawangling, and Jianfengling are distributed in both groups, and the distribution is relatively chaotic. The small subgroups clustered in group I were divided into three small independent subgroups, indicating a large difference in kinship distance between the large group and each other. The aggregation of samples in group II was relatively uniform. Therefore, although there is certain geographical isolation between the *H. hainanensis* resources of different population areas, there is no direct correlation between the clustering based on genetic distance and its geographical source.

#### 3.3.2 Analysis of population genetic structure

**Figure 3:**
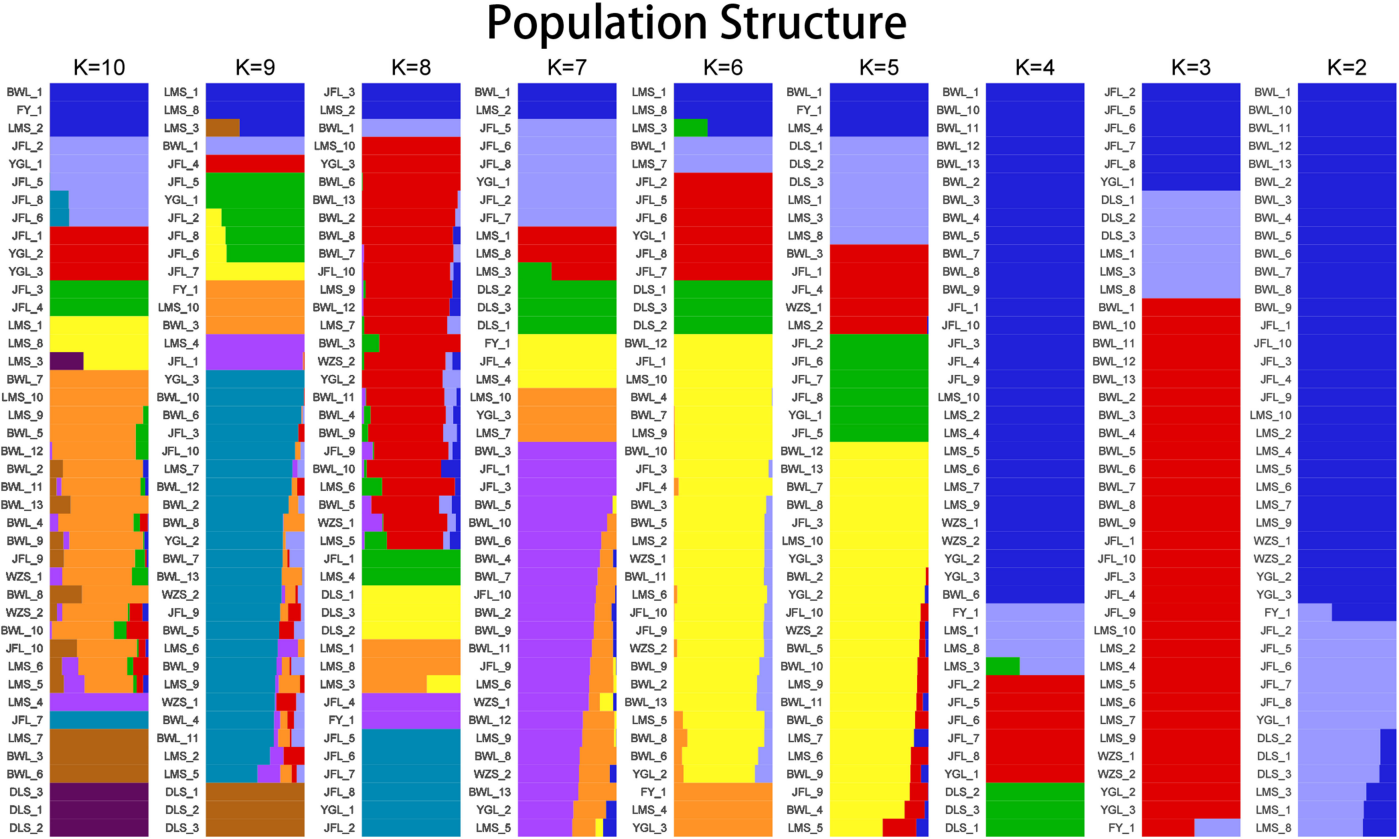
The population structure analysis on *Hopea hainanensis*.

**Figure 4:**
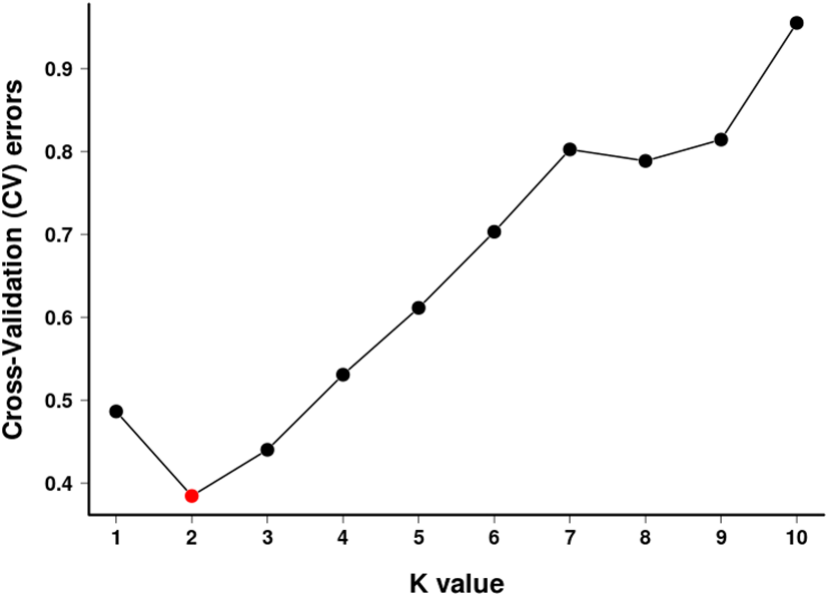
K selection of population structure.

To further verify the evolutionary genetic relationship between the samples and infer that the *H. hainanensis* population likely came from several ancestors. The genetic structure of the mutations in each sequencing sample was further studied. Based on SNP data, ADmixture software was used to analyze the population structure of all samples. Then, cluster analysis was performed, assuming that the number of clusters (K value) was 1-10. Different K values represent the distribution of ancestral genetic material of different populations under the assumption that there are K ancestral groups. Since K=1 cannot represent the distribution of ancestral genetic material of different populations, it is not shown in the figure. As shown in **Figure 3**, when K=2 and the sample are divided into two subgroups, the sample of group 1 is almost dark blue, and the sample of group 2 is almost light purple. The samples from Fanyang (FY), Diaoluoshan (DLS), Jianfengling (JFL), and Limushan (LMS) were clustered into group 2, and the remaining samples were clustered into group 1. In the Cross-Validation (CV) errors graph (**Figure 4**), when K=2, CV error achieves the minimum value, indicating that the genetic differences between samples are relatively large and the genetic relationship is distant. Therefore, it can be preliminarily concluded that the seven *H. hainanensis* populations in Hainan Island came from two different ancestors, and there was less gene exchange among them. In the table of genetic differentiation coefficients among populations (**Table 4**). The F_st_ values among the seven *H. hainanensis* populations ranged from -0.05258 to 0.29542. There was significant genetic differentiation (F_st_ > 0.25) between FY, WZS, and DLS populations. The genetic differentiation between DLS and BWL, WZS populations, and FY and BWL populations was significant (0.15 < F_st_ < 0.25). There was a moderate genetic differentiation between BWL and JFL, YGL, FY and LMS, JFL and DLS, and the other three populations (0.05 < Fst < 0.15). In addition, the genetic differentiation among other populations was low, so differentiation could not be considered (F_st_ < 0.05).

**Table 4:** Genetic differentiation coefficient(F_st_: above diagonal).

	BWL	LMS	JFL	FY	WZS	YGL	DLS
BWL		0.02412	0.07731	0.19692	-0.02501	0.06426	0.19716
LMS			0.03763	0.07358	-0.04653	-0.0011	0.11088
JFL				0.06496	0.00063	-0.03522	0.09211
FY					0.25587	0.02041	0.29542
WZS						-0.05258	0.18954
YGL							0.1024
DLS							

#### 3.3.3 Principal component analysis

**Figure 5:**
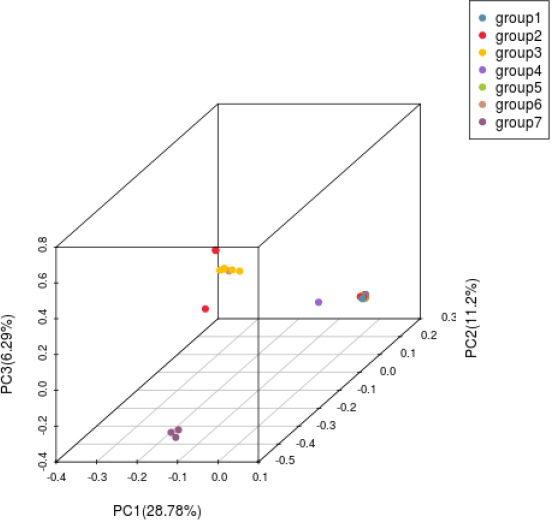
Principal component analysis diagram of *H. hainanensis*.

Principal component analysis (PCA) was carried out on H. hainanensis population samples from Hainan Island to determine the evolutionary relationship among the populations further. When the geographical distance between groups is relatively close, PCA can better reflect the relationship between groups. Samples with similar genetic backgrounds will gather in the figure to form a cluster. The farther the distance between the two samples in the figure, the greater the genetic background difference between the two samples. As shown in the following figure (**Figure 5**), the 42 *H. hainanensis* were clustered to form three independent clusters, among which eight samples from Jianfengling (JFL_2, JFL_5-JFL_8), Limushan (LMS_3, LMS_8) and Yinggeling (YGL_1) populations with similar genetic backgrounds were clustered together to form cluster 1. Fanyang (FY_1), Wuzhishan(WZS_1-WZS_2), Bawangling (BWL1-BWL_13), Yingge Mountain (YGL_2, YGL_3), Jianfeng Mountain (JFL_1, JFL_3-JFL_4, JFL_9-JFL_10) and Limushan (LMS_1, LMS_2, LMS_4-L). The 31 samples from the six populations of MS_7, LMS_9, and LMS_10 were clustered together with similar genetic backgrounds to form cluster 2. The population of DLS_1-DLS_3 was far away from the other 2 clusters, showing a long genetic distance, so the population of DLS_1-DLS_3 formed a cluster alone. 

#### 3.3.4 Analysis of the genetic relationship

**Figure 6:**
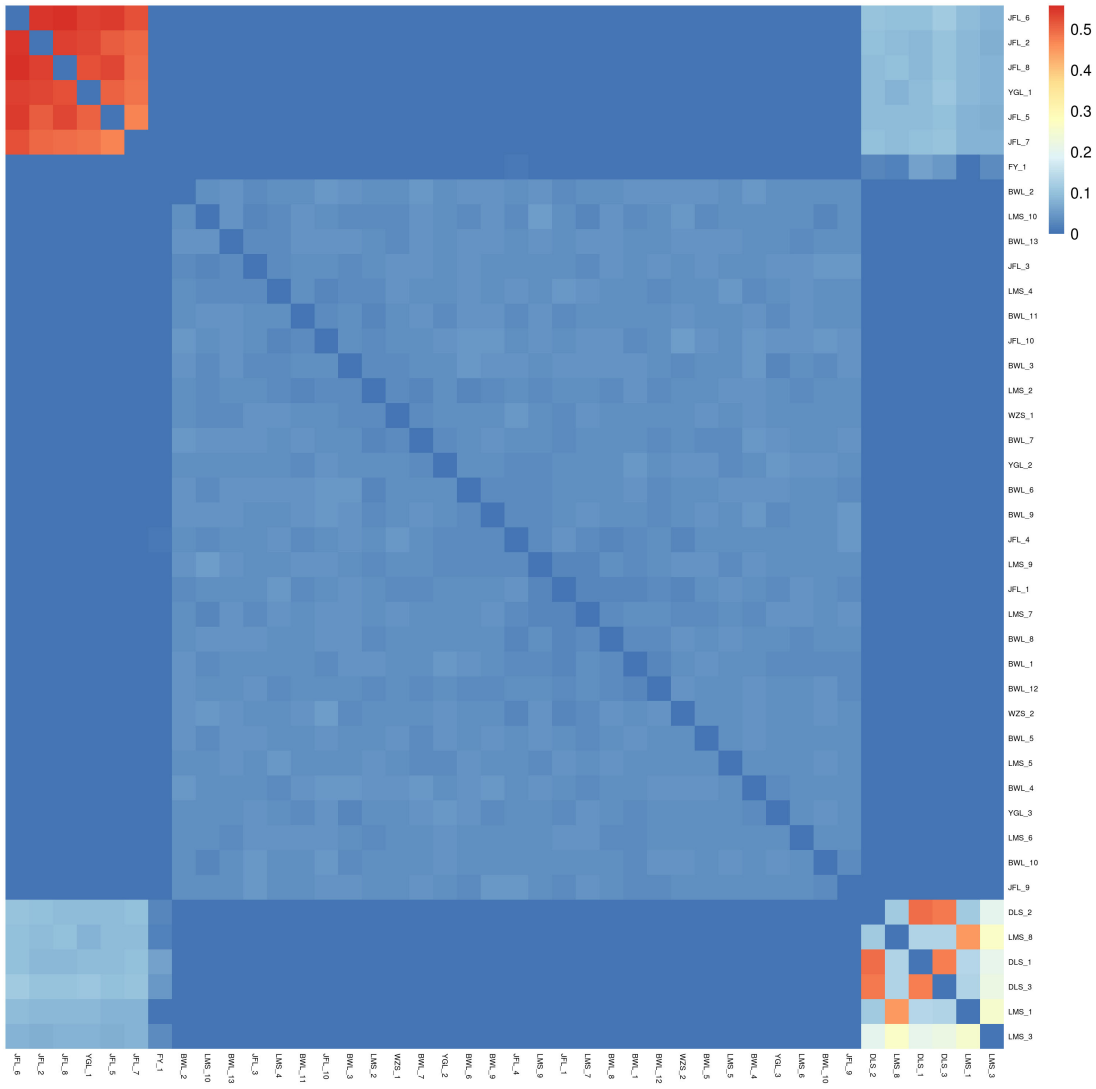
Ties of consanguinity.

In the relatedness heatmap (**Figure 6**), the relatedness coefficient was more significant than 0.4 (between the three samples of DLS_1, DLS_2, and DLS_3). The relatedness among the three samples of Diaoluoshan was very close. The genetic distance between LMS_8 and LMS_1 was very close in the relatedness heatmap. It can be concluded that the samples in the same population are more closely related, and the more distant the geographical location, the more complex the gene exchange, and the more distant the genetic relationship. The six samples, YGL_1, JFL_2, and JFL_5-JFL_8, are closely related. The three samples from Diaoluoshan (DLS_1-DLS_3) and Limushan (LMS_1) are just between 0.2 and 0.3. This indicates that there is still some genetic exchange between clusters under geographical isolation.

## 4 Discussion

### 4.1 Genetic diversity in *Hopea hainanensis*

SNP variation is the most important and widespread type of sequence variation in the plant genome, which can be easily identified by sequence alignment (Fang et al., 2014). In this study, 48795 high-quality SNPs were obtained by screening and filtering. In the natural state of *H. hainanensis* field, the ecological range of the population is wide. The seeds are winged nuts, and the germination rate is high. Still, the seeds have higher requirements for germination conditions in the natural environment, which restricts the development of the population (Trang and Triest, 2019). Even if the *H. hainanensis* seeds can germinate and grow into seedlings in the natural population, the *H. hainanensis* seedlings are easy to be eliminated due to their weak competitiveness, resulting in few remaining adult *H. hainanensis* plants and weak natural regeneration ability of the population in the field (Kenta et al., 2004; Mehmood et al., 2021). The population density was very low, leading to the population’s weak reproductive ability and stress resistance and slow natural recovery and development. Genetic diversity is lost when the effective population shrinks and mating is switched from outcrossing to selfing (Ellegren and Galtier, 2016; Cai et al., 2021). It is most likely that a severe demographic bottleneck is responsible for the low genetic diversity of *H. hainanensis* populations. Over the past 300 years, this species has lost about 70% of its population (Ly et al., 2018). In the 20th century, Hainan Island’s deforestation increased rapidly. About 80% to 95% of the primary forests have been destroyed because of logging for wood on a large scale. Furthermore, transitions to rubber trees and Eucalyptus plantations, and the growth of cities (Lin et al., 2017; Chen et al., 2018; Sun et al., 2020). Due to the high quality of its wood, the number of *H. hainanensis* trees would go down proportionally, or maybe even more. There is a lack of genetic diversity analysis on the endangered mechanism of levees, especially on the genetic diversity of levees in different fragmented habitats.

Based on SNP, simplified genome sequencing analysis was performed on 42 *H. hainanensis* samples using GBS technology. After obtaining the data, genetic evolution and population analysis were performed, such as phylogenetic tree clustering analysis, population genetic structure analysis, principal component analysis, and phylogenetic relationship analysis. In principal component analysis, the contribution rates of the first principal component (PC1), the second principal component (PC2), and the third principal component (PC3) were 28.78%, 11.2%, and 6.29%, respectively. The contribution rates of the three principal components selected in this analysis were all low, and the total contribution rate was less than 50%. Therefore, there may be a deviation (difference) between the cluster results of PCA and the analysis results of other groups. In the principal component analysis, the genetic distance of the Diaoluoshan population was far from the other populations, and a single cluster was formed. Except for principal component analysis, the population structure of all samples, K value selection of population structure, and phylogenetic evolutionary tree analysis results showed that the cluster division was the same and supported the division of seven populations into two populations. Therefore, it is more reasonable to divide the 42 *H. hainanensis* samples from seven populations into two groups: Group 1 (Diaoluoshan, Limu shan, Yinggeling, Jianfengling) and Group 2 (Wuzhishan, Fanyang, Bawangling). In this study, high-throughput GBS sequencing was performed based on SNPs, and the analysis results may be limited due to the lack of reference genomes covering the whole genome of SNP.

### 4.2 Population genetic structure and differentiation in *Hopea hainanensis*

Genetic structure is influenced by many factors, such as breeding system, genetic drift, population size, seed dispersal, gene flow, evolutionary history, and natural selection (Konuma et al., 2000; Mehmood et al., 2020). The terrain of Hainan Island is low, flat ground, and high in the middle. The terrain takes Wuzhishan and Yinggeling as the uplifted core and drops progressively to the periphery. The mountain, hill, platform, and plain form a ring-stratified landform with an obvious cascade structure. The samples collected in this study were taken from Wuzhishan, Yinggeling Mountain, and adjacent forest reserves. In geographical location, the Jianfenglin population and Diaoluoshan population, Limushan between groups are far apart (> 100 km). Still, the smaller the genetic distance between the two groups (F_st_ = 0.09211), the existing gene flow between populations may have originated from the common ancestor of genetic exchange, carried by man-made factors, animals or other factors such as geological factors into the other group.

## 5 Conclusion

In order to improve genetic diversity among *H. hainanensi*s populations, the *H. hainanensis* population resources of endangered plants should be effectively protected and developed. In order to protect *H. hainanensis* species, *H. hainanensis* seedlings may be protected *ex situ* due to their weak competitive ability and easy inhibition by mother trees. By conserving *H. hainanensi*s seedlings *ex-situ*, we can reduce competition within the population and increase competition between poke stack populations. Genetic drift can also be reduced by increasing gene flow among small populations. Additionally, cross-introduction and breeding among the seven populations can improve genetic diversity.

## 6 Implications for conservation

Because the loss of genetic variation is a major threat to endangered species, preserving and restoring genetic variation is an important conservation action (Jiang et al., 2018; Cai et al., 2021). We discovered that genetic variation in the populations BWL, WZS and FY were low. These populations are more vulnerable to biotic and abiotic stresses, their conservation is critical. Furthermore, the populations DLS, YGL, JFL and LMS had higher levels of genetic diversity and contained more than one genetic subgroup. That populations could be used as seed sources for propagating seedlings and saplings in restoring Hainan Island’s previously logged lowland rainforests. It is difficult to regenerate native *H. hainanensi*s populations because seedlings and saplings grow slowly and are frequently unable to establish themselves in heavily shaded conditions. To help restore endangered *H. hainanensis* populations on Hainan Island, select populations with high genetic diversity (e.g., for seedlings).

## Data availability statement

The datasets presented in this study can be found in online repositories. The names of the repository/repositories and accession number(s) can be found in the article/supplementary material.

## Author contributions

Conceptualization, LZ, MMN; methodology, YKC, HLZ, XTZ; software, HLZ, YKC, and MMN; validation, MMN; formal analysis, HLZ, LZ and MMN; investigation, YKC, and XTZ; resources, YKC; data curation, YKC; writing—original draft preparation, LZ, HLZ, HYZ, TTL, XTZ, YKC, and MMN; writing—review and editing, LZ, HLZ, HYZ, TTL, XTZ, YKC, and MMN; visualization, YKC; supervision, YKC; project administration, YKC; funding acquisition, YKC All authors have read and agreed to the published version of the manuscript. The data presented in the study are deposited in the Dryad repository.
