# Corrigendum: Genetic diversity assessment of *Hopea hainanensis* in Hainan Island

**DOI:** 10.3389/fpls.2023.1121759

**Published:** 2023-02-17

**Authors:** Yukai Chen, Hai-Li Zhang, Li Zhang, Mir Muhammad Nizamani, Taoxiu Zhou, Haiyang Zhang, Tingting Liu

**Affiliations:** ^1^ Ministry of Education Key Laboratory for Ecology of Tropical Islands, College of Life Sciences, Hainan Normal University, Haikou, China; ^2^ Hainan Key Laboratory for Sustainable Utilization of Tropical Bioresources, School of Life Sciences, Hainan University, Haikou, China; ^3^ Guizhou Normal University Museum, Guizhou Normal University, Guizhou, China; ^4^ Department of Plant Pathology, Agricultural College, Guizhou University, Guiyang, China; ^5^ College of Biological Science and Technology, Yangzhou University, Yangzhou, China; ^6^ College of International Studies, Sichuan University, Chengdu, China

**Keywords:** conservation biology, endangered tree, hopea hainanensis, SNP, GBS

In the published article, there was an error in Abstract, paragraph one.

This sentence previously stated:

“The results showed that the average heterozygosity of the six populations of *Hopea hainanensis* was 19.77%, which indicated that the genetic diversity of *Hopea hainanensis* was low.”

The corrected sentence appears below:

“The results showed that the average heterozygosity of the seven populations of *Hopea hainanensis* was 19.77%, which indicated that the genetic diversity of *Hopea hainanensis* was low.”

In the published article, there was an error in 1. Introduction, paragraph four.

This sentence previously stated:

“It was identified as endangered species in the Red Book of Chinese Plants. The ramps as “Endangered” by the IUCN (Ly et al., 2018).”

The corrected sentence appears below:

“It was identified as an endangered species in the Red Book of Chinese Plants and is ranked as “Endangered” by the IUCN (Ly et al., 2018)”.

In the published article, there was an error in 1. Introduction, paragraph five.

This sentence previously stated:

“The construction and analysis of genetic maps, the study of genome-wide association systems and gene diversity and identifying the germplasm of plants and animals. Therefore, in this study, P-GBS technology was used to systematically identify 42 genome-wide SNPs of H. hainanensis resources.”,

The corrected sentence appears below:

“These include the construction and analysis of genetic maps, the study of genome-wide association systems and gene diversity, and identifying the germplasm of plants and animals. Therefore, in this study, GBS technology was used to systematically identify 42 genome-wide SNPs of H. hainanensis resources. “.

In the published article, there was an error in 3. Results, 3.3. Genetic evolution and population analysis, 3.3.3 Principal component analysis.

The sentence below should be removed:

“PC1、PC2、PC3 分PC1, PC2, and PC3 represent principal component 1, principal component 2, and principal component 3, respectively”.

In the published article, there was an error in 4. Discussion, *4.1 Genetic diversity in* Hopea hainanensis, paragraph two.

The sentence below should be removed:

“In PCA, the contribution rates of the PC1, PC2, and PC3 were 28.78%, 11.2%, and 6.29%, respectively. The contribution rates of the three principal components selected in this analysis were all low. The total contribution rate is less than 50%, so the results of the PCA cluster may be biased from those of other groups.”

The authors apologize for these errors and state that this does not change the scientific conclusions of the article in any way. The original article has been updated.

